# Paediatric obstructive sleep apnoea syndrome (OSAS) is associated with tonsil colonisation by *Streptococcus pyogenes*

**DOI:** 10.1038/srep20609

**Published:** 2016-02-10

**Authors:** Elisa Viciani, Francesca Montagnani, Simona Tavarini, Giacinta Tordini, Silvia Maccari, Matteo Morandi, Elisa Faenzi, Cesare Biagini, Antonio Romano, Lorenzo Salerni, Oretta Finco, Stefano Lazzi, Paolo Ruggiero, Andrea De Luca, Michèle A. Barocchi, Andrea G. O. Manetti

**Affiliations:** 1 GSK Vaccines S.r.l., Via Fiorentina, 1 53100 Siena, Italy; 2 Department of Medical Biotechnologies, University of Siena, Viale Bracci 16, 53100 Siena, Italy; 3 University Division of Infectious Diseases, Hospital Department of Specialized and Internal Medicine, Viale Bracci 16, 53100 Siena, Italy; 4 Clinica Otorinolaringoiatrica, Policlinico Universitario “Le Scotte”, Viale Bracci 16, 53100 Siena, Italy; 5 Section of Pathology, Azienda Ospedaliera Universitaria Senese, Viale Bracci 16, 53100 Siena, Italy

## Abstract

The involvement of pathogenic bacteria in obstructive sleep apnoea syndrome (OSAS) has yet to be elucidated. We investigated the possible role of group A streptococcus (GAS) in OSAS pathogenesis. In 40 tonsillectomized patients affected by OSAS and 80 healthy controls, significant (*p* < 0.0001) association of GAS with paediatric OSAS was found. Supernatant from streptolysin O (SLO)-producing GAS induced production of cysteinyl leukotrienes (CysLTs) in tonsil mononuclear cells (TMCs). CysLTs-treated TMCs showed significant (*p* < 0.05) proliferation of CD4+ T, CD19+ and CD19+CD27+CD38+ B lymphocytes. We discovered a SLO-dependent activation of CysLTs production through a pathway involving TOLL-like receptor 4 (TLR4), TIR-domain-containing adapter-inducing interferon-β (TRIF), Myeloid differentiation primary response gene 88 (MyD88), and p38 MAP Kinase. In conclusion, we hypothesise that GAS may contribute to paediatric tonsillar hyperplasia through CysLTs production induced by SLO, and this might explain its association with OSAS.

In paediatrics, despite the use of antibiotics, adenotonsillectomy is commonly indicated to treat certain tonsil diseases^1^,^2^,^3^,^4^. Tonsillectomy has been routinely applied to treat recurrent tonsillitis (RT); more recently, obstructive sleep apnoea syndrome (OSAS) has emerged in children, as the primary indication for surgical removal of adenoids and tonsils^1^,^5^,^6^. OSAS has been associated with cardiovascular, growth and neurobehavioral abnormalities, inflammation, and primarily with hypertrophy of the tonsils and adenoids^6^,^7^,^8^,^9^. Adenotonsillar hypertrophy is by far the major pathophysiological contributor to OSAS in children^10^,^11^, and is most commonly measured following the Brodsky scale ranging from 0–4, where 4+ described kissing tonsils^1^.

*Streptococcus pyogenes* (GAS) is a human pathogen causing a wide range of diseases^12^. Although GAS is considered the most common single organism associated with bacterial pharyngo-tonsillitis^13^, it is unclear if antibiotics are necessary to treat asymptomatic infections^14^. Moreover, GAS has never been associated with OSAS, though a possible microbial aetiology for hypertrophy has been hypothesised^15^.

Cysteinyl leukotrienes (CysLTs) are a family of inflammatory lipid mediators synthesized from arachidonic acid by innate immune cells^16^. The CysLTs LTC4, LTD4 and LTE4 and their receptors LT1-R and LT2-R are more abundant in OSAS than in RT paediatric tonsils^7^,^17^. Up-regulation of LT1-R and LT2-R in T and B lymphocytes of children with OSAS could be involved in promoting tonsil enlargement^18^,^19^,^20^. Furthermore, LTD4 mediates proliferative and inflammatory signalling pathways in adenotonsillar tissues from OSAS children^21^.

Interestingly, the secreted GAS thiol-activated toxin streptolysin O (SLO) induces CysLTs synthesis by human granulocytes^22^. Moreover, SLO activates toll like receptor 4 (TLR4)^23^, whose signalling pattern regulates the activation of cytosolic phospholipase A2 (cPLA_2_) in LPS-activated murine macrophages^24^.

In this study, we investigated the association between pathogenic bacteria and OSAS, the involvement of the GAS toxin SLO in the production of CysLTs by human TMCs and the relationship between SLO and tonsil cell proliferation *via* CysLTs action, hypothesising that GAS, through SLO-induced CysLTs, could be involved in tonsil hyperplasia, which in turn might cause OSAS.

## Results

**Figure 1:**
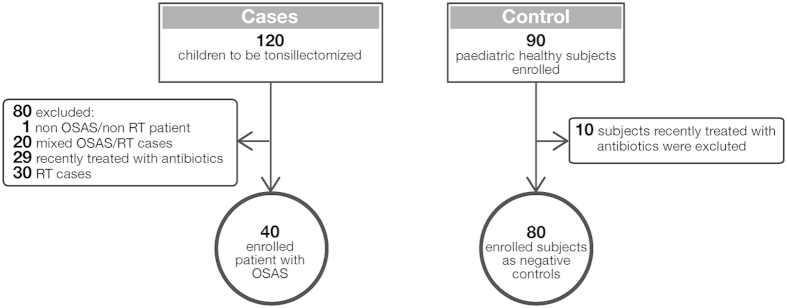
Study design. Children aged ≤16 years. OSAS (obstructive sleep apnoea syndrome); RT (recurrent tonsillitis).

We included 40 tonsillectomized patients affected by OSAS and 80 healthy controls (Fig. 1). OSAS cases were exclusively paediatric (≤16 years) and were matched with the 80 paediatric controls. No significant differences were noted in the demographics of the matched populations (Table 1). None of the patients included in the study were recently (≤10 days) treated with antibiotics before the tonsillectomy or swab.

In the exclusively paediatric OSAS group, we found that GAS was the pathogen most significantly associated with OSAS (OR = 7.14, 95% CI 2.81–18.10, *p* < 0.0001), although *H. influenzae* and *S. pneumoniae* also showed an association, but with a wider confidence interval (Table 2).

As reported in Supplementary Table S1, 22 GAS strains were isolated from the 40 OSAS patients, with co-isolation of different GAS strains from three patients. GAS strains were also co-isolated with other bacteria, typically *S. aureus* (7 OSAS and 4 controls), *H. influenzae* (4 OSAS and 1 control) and *S. pneumoniae* (4 OSAS and 1 control). Notably, M75 and M4 GAS strains were prevalent in the OSAS cohort (31.8% and 13.6% respectively), and were absent in healthy controls, whereas one M18 strain was found in one control but not among the OSAS GAS strains (Supplementary Table S2).

**Figure 2:**
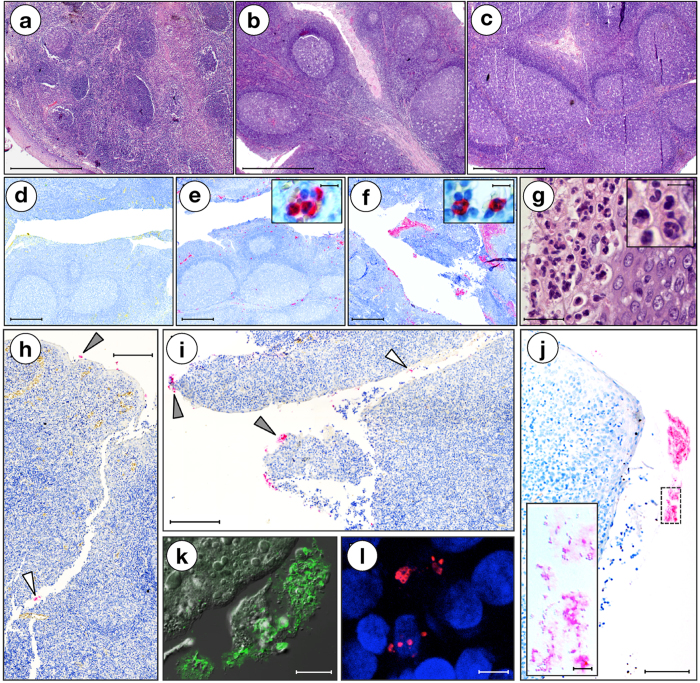
(**a-g)** Follicular hyperplasia grade and neutrophil infiltrate level. Micrographs of each condition of follicular hyperplasia grade and neutrophil infiltrate level are shown and specific areas of e-g micrographs are magnified. (**a**–**c**) specimens representative of scores 1, 2, 3 for germinative centres, respectively. HE staining. Bar = 1 mm. (**d**–**f**) anti-CD15 IHC staining, specimens representative of neutrophil (red) score 0, 1 and 2 respectively. Inserts in E and F show positively-stained neutrophils (red) at higher magnification. Bar = 500 μm; 10 μm in the inserts. (**g**) HE-staining of a specimen representative of neutrophil score 2; insert shows neutrophils at higher magnification. Bar = 50 μm; 10 μm in the insert. (**h, l**) GAS colonization. (**h–j**) IHC micrographs show GAS (red staining) on the surface (grey arrowheads) and the crypts (white arrowheads) of OSAS human tonsils. The insert in (**j)** shows the area delimited by dashed line at higher magnification and using Differential Interference Contrast (DIC). (**k**) Immunofluorescence (IF) + DIC image showing GAS (green) on the surface and in tight contact with the tissue of tonsils from OSAS patients. (**l**) Confocal microscopy representing GAS (red) colonising human tonsils from OSAS. Scale bars for all the micrographs are shown: (**h**, **i)**: bar, 200 μm; (**j**) 100 μm, 5 μm in the insert; (**k**) 20 μm; (**l**) 5 μm.

The histomorphological analysis of OSAS and particularly GAS-positive OSAS tonsils suggested a sub-acute/chronic infection, with hyperplastic germinal centres (GCs) and minimal inflammation (Fig. 2a-g). In fact, over 70% of analysed OSAS presented moderate or high follicular hyperplasia and low or moderate neutrophil infiltrate (Table 3). Notably, over 62% of GAS strains were isolated from these patients. Non-activated GCs or severe neutrophil infiltrate were never found. In OSAS cases, GAS was detected both on the tonsil surface and in the crypts, as chains or small aggregates, suggesting a limited bacterial load (Fig. 2h-j).

Tonsils from pediatric OSAS have an increased CysLTs production, which mediates tonsil cell proliferation^21^,^25^. GAS toxin SLO was found to induce CysLTs production by human granulocytes^22^. We assessed the ability of SLO to induce CysLTs in TMCs of tonsillectomized patients, to investigate whether, and through which signalling pattern, GAS colonisation may be associated with CysLTs production and ultimately with cell proliferation and thus hypertrophy in OSAS tonsils.

**Figure 3:**
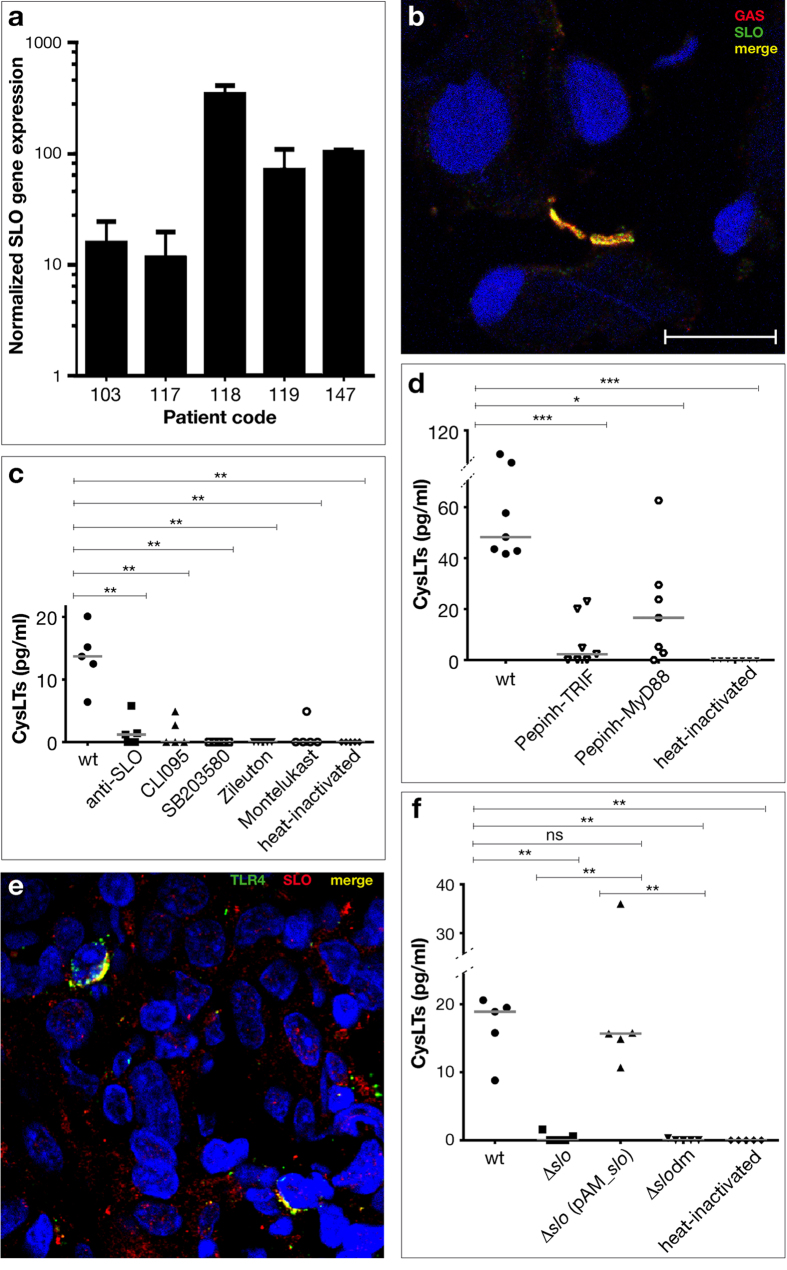
(**a,b)** GAS strains produce SLO in OSAS tonsils. (**a**) *in vivo* expression analysis (real time qRT-PCR), of *slo* in OSAS tonsil tissue. The quantity of cDNA for *slo* gene was normalized to the quantity of *gyrA* cDNA in each RNA sample. The reported values, expressed as fold changes, are the means ± standard errors from three independently isolated RNA preparations analyzed in triplicate. (**b**) Confocal microscopy image shows GAS (red) expressing SLO protein (green) in tonsil tissue of patient 147 (tonsil cell nuclei in DAPI). The merge between anti-GAS and anti-SLO antibodies resulted in a yellow color. Bar, 10 μm. (**c–e**) SLO induces CysLTs production by human TMCs interacting with TLR4 *in vitro* though a TRIF- and MyD88-dependent signaling pathway. (**c,d**) anti-SLO antibodies, CLI 095 (TLR4 inhibitor), SB203580 (p38 MAPK inhibitor), Zileuton (5-LO inhibitor), montelukast (LT1-R inhibitor), Pepihn-TRIF (a TRIF inhibitor), or Pepihn-MyD88 (a MyD88 inhibitor) inhibit production of CysLTs by human TMCs induced by GAS 147 supernatant. (**e**) Confocal microscopy image shows the SLO protein (red) and TLR4 (green) merged (yellow) in two tonsil cells (cell nuclei in DAPI) of patient 147. Bar, 10 μm. (**f**) SLO induces CysLTs production by human TMCs ***in vitro***. Supernatant of GAS strain 3348, but not of the *slo* isogenic mutant (3348Δ*slo*) or of the non-toxic double mutant (3348*slo*dm), induces CysLTs production in human TMCs. Complementation 3348Δ*slo*(pAM_*slo*) restored wt SLO expression and thus the ability to induce CysLTs production. (**c**,**d**,**f**): each dot represents a single donor of human TMCs; grey bars = medians. *p < 0.05, **p < 0.01, ***p < 0.001, Mann-Whitney U-test.

SLO production in tonsil-colonising GAS was demonstrated with real time qRT-PCR using GAS RNA extracted from five selected GAS-positive OSAS tonsils. The *slo* gene was transcribed by each GAS strain analysed. (Fig. 3 panel a). SLO-expressing GAS cells in tonsil tissue were also visualised by immunofluorescence (Fig. 3 panel b). Moreover, western blot analysis of the supernatant of all the GAS-positive OSAS strains, at early stationary phase, showed that all strains produced SLO *in vitro* (data not shown). Thus, we incubated the supernatant of GAS strain 147, containing a sub-lytic concentration of about 200 ng/ml of SLO toxin, with TMCs of tonsillectomized patients, to assess CysLTs production. As reported in Fig. 3 panel c, supernatant of strain 147 induced CysLTs production, which was abolished by heat-inactivation or anti-SLO antibody. Since SLO activates TLR4 in murine bone marrow–derived macrophages^23^, the molecular mechanisms involved in SLO-dependent CysLTs production were dissected using specific inhibitors targeting the TLR4 cell signaling and CysLTs pathway. CLI095 (a TLR4 specific inhibitor)^26^,^27^, SB203580 (p38 MAPK inhibitor)^28^, Zileuton (5-LO inhibitor)^29^, and montelukast (a LT1-R inhibitor)^17^,^21^, inhibited the activity of 147 supernatant, demonstrating the involvement of TLR4, through p38 MAPK, 5-LO and LT1-R, in the process of SLO-induced CysLTs production (Fig. 3 panel c). In addition, CysLTs production was significantly inhibited by Pepihn-TRIF (a TRIF inhibitor), and less significantly by Pepihn-MyD88 (a MyD88 inhibitor)^30^,^31^, demonstrating TLR4-induced CysLTs production through a TRIF- and MyD88-dependent pathway (Fig. 3 panel d).

Immunofluorescence and confocal microscopy of human tonsil tissue showed localisation in the same area of SLO and TLR4 signals, confirming a role for SLO interaction with TLR4 in CysLTs production (Fig. 3 panel e).

To further confirm that CysLTs production was due to the secreted SLO, we used the supernatant of GAS strain 3348, an invasive M1 isolate containing a sub-lytic concentration of approximately 200 ng/ml of SLO toxin. Notably, the isogenic mutant of *slo* (3348Δ*slo*) was strongly impaired in its ability to induce CysLTs production, and the knock-in of a double mutated version of the *slo* gene (3348*slo*dm), lacking toxicity and with severely reduced cell-binding capacity^32^, failed to restore SLO activity, suggesting the involvement of the two mutated sites in the SLO binding to human TMC and to the TLR4 receptor (Fig. 3 panel f). A complemented strain (3348Δ*slo*(pAM_*slo*)), producing wild type SLO, restored the capacity for the induction of CysLTs production (Fig. 3 panel f). The majority of these data were also confirmed using peripheral blood mononuclear cells (PBMCs) obtained from healthy subjects (data not shown).

**Figure 4:**
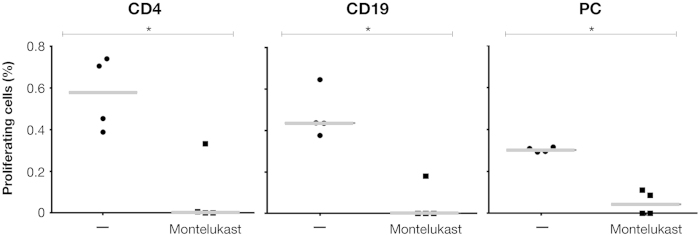
CysLTs LTD4 induces helper T cell and plasma B cell proliferation in human tonsils of OSAS patients. Tonsil mononuclear cells were stimulated with LTD4 (1 nM) alone, or with LTD4 and montelukast (10 μM) at time 0 and analyzed by FACS at day 3 post stimulation, for proliferation of B and T cells. Proliferation was detected for CD4+ (helper T cells), CD19+ (B cells), and CD19+CD27+CD38+ (plasma B cells). Each dot represents human tonsil mononuclear cells of a single OSAS patient, and medians (grey bars) are reported. Statistical analysis was performed using a non-parametric Mann-Whitney U-test (*p < 0.05).

To test the capacity of CysLTs to induce proliferation in tonsil tissue, we used the CysLT LTD4 in a proliferation assay with TMCs. As reported in Fig. 4, LTD4 stimulated the proliferation of helper T cells and plasma B cells, which was significantly inhibited by montelukast, suggesting the involvement of LT1-R in the proliferation process.

**Figure 5:**
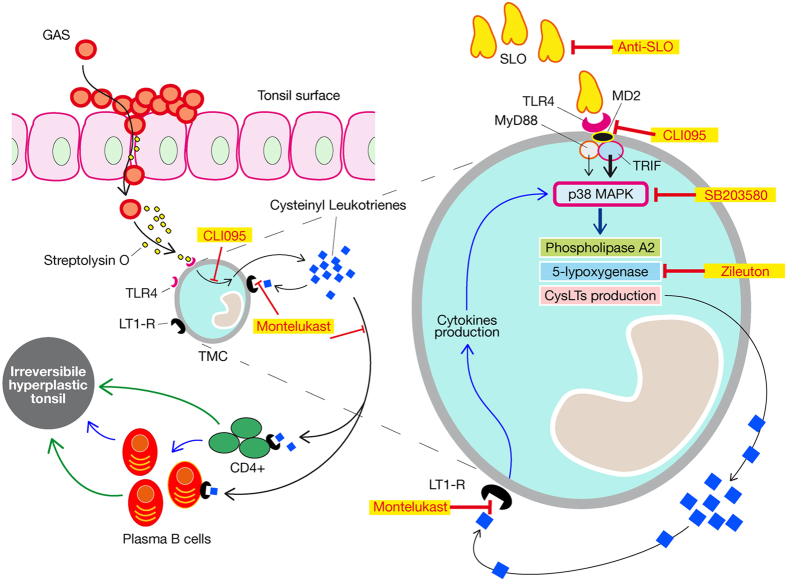
Hypothesized working model for tonsil hyperplasia. OSAS is characterized by an irreversible tonsil hyperplasia. Here we hypothesise on one of the mechanisms causing such pathology. GAS-induced tight junction loosening and epithelial damage allow bacteria and/or bacterial virulence factors penetrating the tonsil tissue. GAS colonising tonsillar tissue of OSAS patients produces SLO protein, which interacting with TLR4, induces the production of CysLTs by TMCs. SLO, binding to TLR4, activates the TRIF- and the MyD88-dependent signaling pathway, which respectively to a major (tick arrow) and minor extent (thin arrow) induce p38MAPK activation, resulting in the phosphorylation of cPLA_2_, which in turn activates the cascade leading to cysteinyl leukotriene production. This production can be further enhanced by the up-take of CysLTs by its specific receptor 1 expressed by the TMCs involved. The CysLTs produced induce in turn, again *via* LT1-R expressed by T and B cells, proliferation of tonsillar helper T cells and plasma B cells, leading to the hyperplasia of the tonsils. The mechanism of cell proliferation could be triggered by the direct interaction of CysLTs with receptor 1 both in T and in B cells (green arrows), or by interaction of CysLTs with receptor 1 in T cells, which, when proliferating, could produce cytokines causing plasma B cell proliferation (blue arrows), which would be responsible for the hyperplasia of the germinal centers described in OSAS-GAS cases.

These results demonstrate that tonsil-colonising GAS produces SLO, which, by interacting with TLR4, induces production of CysLTs by human innate immune cells. SLO, binding to TLR4, activates the TRIF- and MyD88-dependent signalling pathway, which by inducing p38 MAPK activation, provokes the phosphorylation of cPLA_2_, which in turn provides the arachidonic acid to be oxidized by 5-LO, thereby activating the cascade leading to CysLTs production. This production might be further enhanced by the uptake of CysLTs by their specific receptor, LT1-R, which is expressed by the innate immune cells involved. CysLTs in turn, induce proliferation of tonsillar helper T cells and plasma B cells, *via* the LT1-R expressed by T and B cells, possibly leading to tonsil hyperplasia. The mechanism of cell proliferation could be triggered by direct interaction of CysLTs with LT1-R of both T and B cells (Fig. 5, green arrows), or only of T cells, which in turn, could produce cytokines causing plasma B cell proliferation (Fig. 5, blue arrows), eventually leading to hyperplasia of the GCs described in GAS-positive OSAS cases (Fig. 5).

In conclusion, we hypothesise that GAS sub-acute chronic colonisation of tonsils may contribute to tonsillar hyperplasia *via* a SLO-dependent TLR4-mediated, TRIF and MyD88-dependent p 38 MAPK pathway which activates CysLTs production, and that this mechanism could be associated with paediatric OSAS disease.

## Discussion

Recently, OSAS has emerged in children as a primary indication for the surgical removal of adenoids and tonsils^1^,^5^,^6^. Chronic tonsillar hyperplasia is a major risk factor for paediatric OSAS, and to date, pathogenic bacteria in tonsils have not been associated with this. Reports indicate that production of CysLTs increases in the tonsils of paediatric OSAS^9^,^17^, and it is known that GAS SLO induces production of CysLTs in human granulocytes^22^. We found that patients who presented with OSAS were more likely to be colonised with GAS. We demonstrate that GAS SLO induces production of CysLTs by human TMCs *via* a TLR4-mediated, TRIF- and MyD88-dependent p38 MAPK pathway, and that the CysLT LTD4 mediates helper T cell and plasma B cell proliferation in tonsils. Thus, we propose a mechanism for human chronic tonsil hyperplasia, and therefore possibly for OSAS, which involves colonisation of tonsil tissue by GAS.

In this prospective case control study, we aimed to investigate the pathology of OSAS tonsils compared with control throat swabs of age-matched healthy subjects. Although several authors have highlighted the presence of pathogenic bacteria in tonsils of patients affected by RT^33^, to our knowledge, no association has been proposed with chronic tonsillar hyperplasia or OSAS to date. Recently, *H. influenzae* and *S. pneumoniae* bacterial biofilms were detected in adenoids of children with OSAS and chronic otitis media, establishing an epidemiological link between these two pathologies^9^.

GAS is associated with acute pharyngotonsillitis^13^, and has been isolated from tonsils of patients affected by RT and tonsillar hyperplasia^2^. However, in the literature, GAS has never been associated with sleep disordered breathing (SDB) diseases such as OSAS. In this study, we found a significant association between OSAS, and GAS colonisation^33^, most of the M-types being M75 and M4, the former infrequently isolated both globally, and in this specific region^34^,^35^,^36^,^37^.

The histopathological analysis of OSAS tonsils revealed a low level of neutrophils, accounting for a sub-acute/chronic infection, and activated GCs compatible with chronically hypertrophic tonsils and thus with OSAS. We isolated GAS from ~50% of these tonsils, revealing bacteria in the epithelial surface and/or in the crypts, where hidden bacterial aggregates of chronically infected tonsils could resist antibiotics and host defences^3^. GAS cells occurred in characteristic chains and/or in small aggregates suggesting a low bacterial load.

Our findings prompted us to consider an alternative mechanism of GAS pathogenesis in tonsils, which could be responsible for chronic infection. OSAS diseases are usually due to adenotonsillar hypertrophy^7^,^9^. Moreover, tonsils from paediatric OSAS have an increased production of CysLTs^9^,^17^, that in turn mediates tonsil cell proliferation in OSAS patients^21^,^25^. As shown, SLO is expressed by GAS strains infecting the OSAS tonsils, and can induce CysLTs production in human TMCs. Notably, other thiol-activated toxins such as alveolysin from *Bacillus alvei*, and theta toxin from *Clostridium perfringens* were reported to generate CysLTs from human granulocytes^22^. The time needed for pore-formation by SLO is between 15 and 20 min at 37 °C. However, a concentration of SLO between 20 and 500 ng/ml is not considered lethal for nucleate mammalian cells, which under these conditions, resealed the pores^38^,^39^. We show that SLO activates TLR4 of mononuclear cells as suggested by the inhibition of CysLTs production in the presence of CLI095, as was also reported previously^23^. TLR4 activation in turn regulates CysLTs production^40^, *via* a TRIF- and MyD88 dependent p38 MAPK pathway, which activates the arachidonic acid cascade^24^, as demonstrated by the inhibition of CysLTs induction in the presence of TRIF, MyD88, MAPK p38 and 5-LO inhibitors. This signalling pathway shows similarities with the LPS pathway, which induces CysLTs in a TRIF- and MyD88-dependent manner and activates cPLA_2_, for the rapid generation of leukotrienes, through the post-traslational mechanism of cPLA_2_ phosphorilation^24^. However, in SLO-dependent CysLTs production, the TRIF-dependent pathway seems more relevant than MyD88, resembling the TRIF-biased signalling described for synthetic monophosphoryl lipid A^41^,^42^. CysLTs production is further enhanced by the interaction with its specific receptor LT1-R, present in several cell types of TMCs as granulocytes, mast cells, monocytes, macrophages and dendritic cells^16^. This is inhibited by montelukast, as demonstrated in our report. Notably, it has been shown, that LT1-R, once activated by CysLTs binding, induces the re-activation of p38 MAPK leading to new CysLTs biosynthesis^43^. Interestingly, the substitution of two specific amino acids in the SLO protein blocked the capacity of GAS to induce CysLTs production. This mutated SLO is non-toxic and was impaired in binding to eukaryotic cells. LTD4 induces proliferation of adenotonsillar cells^21^,^25^, again *via* LT1-R, whose expression was found to be enhanced in peripheral T lymphocytes of OSAS patients as compared to controls^9^,^18^. We have shown that tonsil cell proliferation, induced by LTD4, specifically stimulates helper T cells and plasma B cells, and may promote the hyperplasia of the tonsils, as postulated previously^44^. The hyperplasia of the GCs described in GAS-positive OSAS cases might be triggered by LTD4 interacting with LT1-R expressed in helper T cells, which, once activated, would produce cytokines causing plasma B cell proliferation in GC. Alternatively, the hyperplasia could be caused by T and B cell proliferation triggered by the direct interaction of CysLTs with LT1-R both in T and in B cells.

The proposed model for tonsil hypertrophy accounts for a CysLTs-mediated action; however, tonsils are the inductive sites for both cell-mediated and humoral immune responses, and thus infection of the tonsils may lead to a reversible hypertrophy of lymphoid tonsillar tissue. In this respect, hypertrophy is part of the physiological immunological reaction^8^. In fact, we found that the frequencies of plasma cells producing GAS-specific IgGs were significantly higher (*p* < 0.05) in GAS-positive than in GAS-negative OSAS patients (data not shown).

The SLO protein is also present in GAS strains isolated from swabs taken from healthy tonsils, which were not hyperplastic. However, we do not know if these strains are intermittent or even quiescent, or if the healthy status of these children is destined to vary. In this case, SLO detection would represent a potential early diagnostic tool. In this regard, we cannot rule out the hypothesis that other GAS virulence factors, through this or other mechanisms, could be involved in tonsil hyperplasia. However, it has to be taken into account that other possible causes for OSAS have been already described, such as obesity^1^. Furthermore, we can’t rule out the hypothesis that GAS, rather than being the etiological agent of OSAS, is its consequence; in other words, the conditions created by OSAS might favour GAS colonization, thus explaining the association found.

Our data, which originate exclusively from pathogenic bacteria, refer to a limited number of cases and are from a single centre in Italy, and thus not necessarily generalizable to other health-care settings. Ideally our studies should now be replicated in a large independent, multicentre cohort.

In conclusion, we hypothesise that GAS sub-acute chronic colonisation of tonsils may contribute to tonsillar hyperplasia via a SLO-dependent TLR4-mediated, TRIF and MyD88-dependent p 38 MAPK pathway which activates CysLTs production, and that this mechanism, although still requiring evidence of cause-effect relationship, could possibly be associated with paediatric OSAS disease.

## Methods

### Study design, patients, and clinical procedures

Between October 2009 and December 2013, we performed a prospective case-control study on 120 paediatric patients admitted for tonsillectomy to the Otorhinolaryngology Unit of the University Hospital of Siena (Siena, Italy). The control group consisted of 80 age- and sex-matched healthy volunteers. Age and sex matched controls were recruited among children attending outpatients services of General and Surgical Paediatric Clinic of Siena hospital in absence of any sign and symptoms of respiratory diseases and/or OSAS and/or recurrent pharyngitis. Children were attending services for: follow up visits for epilepsy, urinary tract infections, mild neurological delay, and programmed anesthesiological visit for minor surgery such as phimosis, undescended testicle and inguinal hernia. However, after consent of parents, before final recruitment, we performed anamnestic and clinic investigation to finally check feasibility for enrolment as healthy controls. Eligible tonsillectomized patients were clinically stable children (aged ≤16 years) affected by OSAS. Patients with airway obstruction and clinical features of OSAS (e.g. intermittent breathing pauses, heavy snoring, and daytime sleepiness) due to severe palatine tonsil hypertrophy^45^, were included in the OSAS group. Clinical diagnosis was confirmed by preoperative evaluation of the patients’ medical histories based on specific questions to the parents/caregiver and on physical examinations. Exclusion criteria were: I) antimicrobial treatment within 10 days before surgery; II) coexisting chronic cardiac, hepatic, renal or pulmonary diseases; III) acquired or congenital immunodeficiency; IV) functional or anatomical asplenia; V) systemic corticosteroids therapy; VI) diabetes; VII) craniofacial syndromes; VIII) neuromuscular disorders or IX) cranial nerve palsies. Control subjects underwent the same exclusion criteria in the absence of any history of previous RT and/or OSAS. Before surgery, otorhinolaryngologists completed the clinical anamnestic card for each patient, drew 4-10 ml of blood, and performed superficial and deep tonsil swabs from anesthetised patients. Tonsillectomies were performed under general anaesthesia by cold knife dissection with electro-cautery. Immediately after surgery, tonsils were aseptically divided into portions for microbiology, cryopreservation, fixation, or for purification of tonsil mononuclear cells. For ethical reasons, healthy subjects underwent only superficial swabs. Notably, the presence of GAS in tonsil core samples of OSAS patients was confirmed in the 84% of the superficial swabs of the same patients. Moreover, an identical microbiological content, between superficial swabs and tonsil core samples, was found in 63% of all the patients. Thus, superficial swabs from healthy subjects were considered as a suitable control.

### CysLTs production assay

We resuspended TMCs cells in DPBS (Invitrogen) with CaCl_2_ 1 mmol/L^22^ at 25 × 10^6^ cells per mL and plated 400 μL cells per well on a 24-well plate. We stimulated TMCs with 100 μL of cell-free supernatants of strains 3348, 3348Δ*slo*, 3348Δ*slo*(pAM_*slo*), 3348*slo*dm, and 147 (OSAS tonsil strain, this study). 100 μL of heat-inactivated bacterial supernatants were used as negative controls. The cells were also treated with 147 supernatant having been previously exposed for 15 min to a 1:250 dilution of rabbit polyclonal anti-SLO. 147 was also used to test TMCs previously incubated for 45 min at 37 °C with 5% CO_2_ with 10 μmol/L montelukast sodium hydrate (Sigma-Aldrich)^46^, or alternatively with 3 μmol/L CLI095 (InvivoGen)^47^, or 25 μmol/L SB203580 (Sigma-Aldrich)^28^, or 10 μmol/L Zileuton (Sigma-Aldrich)^29^. TMCs were also previously incubated for 6 h at 37 °C with 5% CO_2_ with the addition of 40 μmol/L Pepinh-TRIF^48^, or 40 μmol/L Pepinh-MyD88 (InvivoGen) following the manufacturer’s instructions and then exposed to 147 supernatant as described earlier. Cells were incubated with bacterial supernatants for 15 min at 37 °C with 5% CO_2_; then the medium was removed by aspiration and the cell supernatants were stored in single-use aliquots at minus 20 °C. CysLT production was assessed using the Cysteinyl leukotriene ELISA kit (Enzo Life Sciences).

### Total RNA isolation, cDNA synthesis, and real-time qPCR

*In vivo slo* gene expression in OSAS tonsils was assayed by real-time quantitative RT-PCR. Total RNA was purified from three stocks of ten 10 μm-thick sections, obtained from 5 GAS-positive frozen OSAS tonsils. We performed three 30s disruptions of tonsil slices in 1 ml TRIzol (Invitrogen), using Lysing Matrix B (MP Biomedicals; Solon, OH) in a Fast Prep FP210 Homogenizer (MP Biomedical) with speed setting 6.5^49^ Samples were incubated on ice between each disruption. After 5 min incubation at rt (room temperature), we centrifuged samples at 9,000× *g* for 1 min. Total RNA in TRIzol was isolated with Direct-zol RNA miniprep (Zymo research) according to the manufacturer’s recommendations, treated with 20 U of Turbo DNase (Ambion), and incubated at 37 °C in a thermal bath for 30 min. Samples were then purified and concentrated with RNA clean & concentrator-5 (Zymo research). We estimated RNA concentration with the ND-1000 Spectrophotometer (NanoDrop Technologies, Wilmington, DE, USA), and performed quality control with the Agilent Bioanalyzer using the RNA 6000 Nano LabChip® Kit (Agilent Technologies, Waldbronn, Germany). We designed primers for *slo* amplification (forward: 5’-SLORT_F: TGCGGGTGTCAATAACAGAA-3’ and reverse: SLORT_R: 5’-TGTCCCAACGTCGTTTTGTA-3’) from *S. pyogenes* M1 strain SF370 *slo* sequence in GenBank (http://www.ncbi.nlm.nih.gov/; NC_002737.1, Gene ID: 900490) using the software Primer3 (http://simgene.com/Primer3). Previously validated reference *gyrA* gene expression (primers: forward: *gyrA*RT_F: 5’-CGACTTGTCTGAACGCCAAA-3’ and reverse *gyrA*RT_R: 5’-TTATCACGTTCCAAACCAGTCAA-3’) was used as the endogenous control for normalization of the data^50^. Amplification efficiency was established for each of the genes from serial dilutions of *S. pyogenes* genomic DNA. The reactions were performed in a Light Cycler 480 II (Roche) using the Light Cycler RNA amplification kit SYBR green I (Roche) according to the manufacturer’s instructions. Each gene was analysed in triplicate and results were evaluated using Light Cycler® 480 SW 1.5 software (Roche). All reactions amplified a single product as determined by melting curve analysis. The expression of *slo* gene as compared with the reference gene *gyrA* expression was evaluated with the relative quantification method (ΔΔCT-Method). Data were represented as relative amounts of mRNA normalized to a *gyrA* control.

#### Tonsil mononuclear cell proliferation assay

TMCs proliferation was assessed by 5-ethynyl-2’-deoxyuridine (EdU) incorporation using the Click-iT-EdU Alexa Fluor 488 cell proliferation assay kit, and specific proliferating TMCs subsets were identified using FACS analysis. Briefly, cells were stimulated with LTD4 alone or with LTD4 and montelukast at time 0 and analysed by FACS 3 days post-stimulation, for proliferation of CD4+ (helper T cells), CD8+ (cytotoxic T cells), CD19+ (B cells), CD19+CD27-CD38- (naive B cells), CD19+CD27+CD38- (memory B cells) and CD19+CD27+CD38+ (plasma B cells). TMCs from 4 tonsils were seeded in 96-well round-bottom Corning® Costar® cell culture plates at 2 × 10^5^ cells per well in RPMI 1640 with 10% FBS (Gibco), 100 U/mL Pen Strep (Life Technologies), 0.05 mg/mL gentamicin, 1X Fungizone® Antimycotic. 10 μmol/L montelukast sodium hydrate was added to dedicated wells for 30 min at 37 °C with 5%CO_2_. After that, 1 nmol/L of Leukotriene D4 (LTD4, Cayman chemical) was added to the wells (with or without montelukast). One well was left untreated as negative control and one was treated with 1μg/mL mouse anti human CD3 and anti-human CD28 as positive control. The plate was incubated at 37 °C with 5% CO_2_ for 72 h. The EdU solution (Invitrogen) was added to the cells during the last 16 h of the 72-h incubation period. We performed FACS analysis after EdU incorporation as previously described^51^. Briefly, we washed the wells with PBS, added Live/dead Near-IR (Invitrogen) and incubated the plate for 20 min at rt in the dark. Plates were washed and pre-mixed mouse antibodies diluted in PBS with 1% BSA (blocking solution) were added for 20 min at rt in the dark. The mouse antibodies were: anti-human CD19 BV605-conjugated (BD Pharmingen), anti-human CD38 BV421-conjugated (BioLegend), anti-human CD44 PerCP-Cy5.5 (eBioscience), anti-human CD10 APC-conjugated (BioLegend). After two washes, cells were incubated with Cytofix/Cytoperm (BD Biosciences) for 15 min at rt in the dark. Cells were washed, then treated with blocking solution with 1% Saponin (permeabilization buffer) and incubated in this solution for 30 min at rt in the dark. Plates were washed, and then cells were resuspended in click-iT reactions and incubated for 30 min at rt in the dark. Cells were washed twice in permeabilization buffer, and then pre-mixed mouse antibodies were added and incubated for 20 min at rt in the dark. Mouse antibodies were: anti-human CD4 PE-Cy5-conjugated (BD Pharmingen), anti-human CD8 BV510-conjugated (BioLegend), and anti-human CD27 PE-conjugated (BD Pharmingen). Plates were incubated for 20 min at rt in the dark. After one wash in permeabilization buffer and one in PBS, FACS analysis was performed on BD LSR Fortessa cell analyzer.

#### Statistics

Normally distributed data are presented as mean with standard deviation (SD); non-parametric data are presented as median. Data were analysed using Mann-Whitney U-test (Fig. 3 and Fig. 4), Z-test (Table 1 sex data; Table 2), or non-parametric Kolmogorov-Smirnov (Table 1 age data). For all analyses, *p* < 0.05 was considered significant. Analyses were done with R and GraphPad Prism 6 software.

#### Ethical Declarations

All clinical investigations were conducted according to Declaration of Helsinki principles. The study was approved by the research ethics committee of the University Hospital of Siena. In more detail, we performed a prospective case-control study on 120 paediatric patients admitted for tonsillectomy to the Otorhinolaryngology Unit of the University Hospital of Siena (Siena, Italy). The control group consisted of 80 age- and sex-matched healthy volunteers. All the parents of all the paediatric subjects involved in the study provided written informed consent.

#### For methods regarding:

*Purification of human peripheral blood mononuclear cells (PBMCs); Tonsil tissue preservation and purification of TMCs; Isolation and characterization of bacterial strains; Bacterial mutant strains, media and growth conditions; Histomorphological analysis and immunofluorescence on FFPE sections; Immunofluorescence on frozen sections* and *Western blot assay,* please consult Supplementary Methods S3.

## Additional Information

**How to cite this article**: Viciani, E. *et al*. Paediatric obstructive sleep apnoea syndrome (OSAS) is associated with tonsil colonisation by *Streptococcus pyogenes. Sci. Rep.* **6**, 20609; doi: 10.1038/srep20609 (2016).

## Supplementary Material

Supplementary Information

## Figures and Tables

**Table 1:** Demographics of the study population stratified by OSAS (obstructive sleep apnoea syndrome), and matched with healthy controls without OSAS (Control).

Characteristics	OSAS	Control	*p* value
**N. patients**	40	80	–
**Age, years**	6 (5–8) (4–12)	7 (4-10) (1–16)	0.3
**Sex, male**	25 (62.5%)	46 (57.5%)	0.59

Age: median (interquartile range [IQR]) and (minimum age - maximum age). Sex: male numbers (%). Statistical analysis was performed using a non-parametric Kolmogorov-Smirnov test on the distribution of age data, and a Z-test on the proportion of cases and controls for sex data (significance level of the test = 0.05).

**Table 2:** Distribution of the studied microorganisms isolated from OSAS patients, and from matched healthy controls without OSAS (Control) expressed in terms of OR. Statistical analysis was performed using a Z-test on the logarithm of the OR (significance level of the test = 0.05).

Organism	Exposed		OR	95%CI	*p* value
	OSAS (n = 40)	Control (n = 80)			
*S. pyogenes*	19 (47.5%)	9 (11.2%)	7.14	2.81–18.10	<0.0001
*S. aureus*	15 (37.5%)	28 (35.0%)	1.11	0.51–2.45	0.79
*H. influenzae*	8 (20.0%)	2 (2.5%)	9.75	1.96–48.45	0.005
*S. pneumoniae*	7 (17.5%)	2 (2.5%)	8.27	1.63–41.94	0.001
*M. catarrhalis*	3 (7.5%)	0 (0.0%)	NC	NC	NC
*S. intermedius*	2 (5.0%)	0 (0.0%)	NC	NC	NC
*S. constellatus*	1 (2.5%)	1 (1.2%)	2.03	0.12–33.25	0.62
*S. agalactiae*	1 (2.5%)	0 (0.0%)	NC	NC	NC
*S. equisimilis*	0 (0.0%)	1 (1.2%)	NC	NC	NC
*P. aeruginosa*	0 (0.0%)	1 (1.2%)	NC	NC	NC

**Table 3:** Total OSAS cases and GAS positive OSAS cases stratified for follicular hyperplasia score against neutrophil infiltrate score.

		Follicular hyperplasia score			
		1	2	3	
Neutrophil infiltrate score	**0**	0/34 (0)	6/34 (17.6)	5/34 (14.7)	n. (%) OSAS
		0/16 (0)	2/16 (12.5)	2/16 (12.5)	n. (%) GAS
	**1**	1/34 (2.9)	6/34 (17.6)	7/34 (20.6)	n. (%) OSAS
		0/16 (0)	3/16 (18.7)	3/16 (18.7)	n. (%) GAS
	**2**	0/34 (0)	5/34 (14.7)	4/34 (11.8)	n. (%) OSAS
		0/16 (0)	3/16 (18.7)	3/16 (18.7)	n. (%) GAS

Scores for each condition based on observation were assigned and reported: absent = 0, low = 1, moderate = 2, high = 3. Total number of analysed OSAS = 34. Total number of isolated GAS in the analysed OSAS = 16.
