# Supplementary material for: Paediatric obstructive sleep apnoea syndrome (OSAS) is associated with tonsil colonisation by Streptococcus pyogenes
Source: Sci Rep. 2016 Feb 10;6:20609. doi: 10.1038/srep20609 (PMC4748291; doi:10.1038/srep20609)
Supplement: Supplementary Information [file srep20609-s1.pdf]

SUPPLEMENTARY INFORMATION

Paediatric obstructive sleep apnoea syndrome (OSAS) is associated with tonsil colonization by *Streptococcus pyogenes*

Elisa Viciani, Francesca Montagnani, Simona Tavarini, Giacinta Tordini, Silvia Maccari, Matteo Morandi, Elisa Faenzi, Cesare Biagini, Antonio Romano, Lorenzo Salerni, Oretta Finco, Stefano Lazzi, Paolo Ruggiero, Andrea De Luca, Michèle A. Barocchi, Andrea G. O. Manetti.

Supplementary Table S1

Bacterial species	OSAS n. (%)	Controls n. (%)
GAS	8 (36.4)	4 (44.5)
GAS + *S. aureus*	3 (13.7)	2 (22.2)
GAS + GAS	2 (9.2)	0 (0)
GAS + *H. influenzae*	2 (9.2)	0 (0)
GAS + *S. aureus* + *S. pneumoniae*	1 (4.5)	1 (11.1)
GAS + GAS + *S. aureus* + *S. pneumoniae*	1 (4.5)	0 (0)
GAS + *S. aureus* + *M. catarrhalis*	1 (4.5)	0 (0)
GAS + *S. aureus* + *H. influenzae*	1 (4.5)	1 (11.1)
GAS + *S. pneumoniae*	1 (4.5)	0 (0)
GAS + *H. influenzae* + *S. pneumoniae*	1 (4.5)	0 (0)
GAS + *M. catarrhalis*	1 (4.5)	0 (0)
GAS + *S. equisimilis*	0 (0)	1 (11.1)
Total	22 (100.0)	9 (100.0)

Supplementary Table S1: List of potentially pathogenic microorganisms co-isolated with GAS from OSAS patients, and from matched controls.

Supplementary Table S2

emm type	OSAS n. (%)	Controls n. (%)
emm 75	7 (31.8)	0 (0)
emm 4	3 (13.6)	0 (0)
emm 89	3 (13.6)	1 (11.1)
emm 3	2 (9.1)	1 (11.1)
emm 12	2 (9.1)	1 (11.1)
emm 1	1 (4.5)	1 (11.1)
emm 5	1 (4.5)	3 (33.4)
emm 28	1 (4.5)	1 (11.1)
emm 77	1 (4.5)	0 (0)
emm 87	1 (4.5)	0 (0)
emm 18	0 (0)	1 (11.1)
Total	22 (100.0)	9 (100.0)

Supplementary Table S2: Distribution of *emm* types among GAS strains isolated from OSAS patients and from matched controls.

Supplementary Methods S3

Tonsil tissue preservation and purification of TMCs. From each specimen, two portions were embedded in OCT compound, plunged in dry ice-cooled isopentane for 1 min and kept frozen at -80°C; two portions were fixed in 4% buffered formaldehyde and paraffin-embedded (FFPE). The remnant portions were single-cell mechanically dissociated, mononuclear cells were purified by Ficoll-Paque Plus (GE Healthcare) following the manufacturer's instructions, and were then treated with RBC (red blood cell) Lysis buffer 1X for 2 min on ice and washed. Cells were counted and resuspended in fetal bovine serum (FBS, Sigma-Aldrich) with 10% DMSO, pre-frozen at -80°C with a Nalgene Cryo freezing container, and stored at -150°C.

Antibody production. Recombinant SLO protein was purified as previously described [1]. Specific antisera were obtained by immunizing CD1 mice intraperitoneally on days 0, 21, and 35 with 10 μg of recombinant SLO plus aluminium hydroxide, or with heat-inactivated GAS bacteria Rabbit (New Zealand) sera were obtained with the same immunogens and schedule, but using 50 μg of SLO and subcutaneous injectionAnimals were bled out on day 49.

Tonsil swabs and cores underwent microbiological analysis. Each specimen was plated on Columbia Agar with 5% sheep blood (COL) and selective agar plates: Columbia CNA agar (CNA), *Haemophilus* selective agar (HAEM-B), Thayer-Martin *Neisseria* selective agar (TM/VCA3), McConkey agar (MCK) and Mannitol salt agar (CHAP) (Oxoid, Basingstoke, UK). All plates were incubated overnight at 37°C: CNA, HAEM-B and TM/VCA3 in a 5% CO_2 enriched atmosphere while COL, HAEM-B, MCK and CHAP under aerobic conditions. Overnight cultures were identified on the basis of macro- and microscopic morphology and identity was confirmed through specific biochemical tests, according to standard methods [2]. Glycerol stocks were made for each bacterial strain.

Bacterial mutant strains, media and growth conditions. The *Streptococcus pyogenes* M1 strain 3348 was kindly provided by Istituto Superiore di Sanità (Rome, Italy); its isogenic mutants 3348Δ*slo*, and 3348*slo*dm were provided by Cristina Faralla and Robert Janulczyk (GSK Vaccines, Siena, Italy). The complemented strain 3348Δ*slo*(pAM_*slo*) was obtained following a previously described method [3, 4] by electro transformation of electrocompetent 3348Δ*slo* strain cells. Briefly, the following primers were used for the *slo* gene insert:

*slo*CompF, forward: 5'-CTGACTGAGCGGCCGCATG TCTAATAAAAAAACATTTAAAAA-3' and *slo*CompR, reverse: 5'-CTGACTGAAGATCTCTAC TTATAAGTAATCGAACCATATG-3'. The restriction digestion of the insert and the pAMp80 plasmid was performed with *NotI* and *BglII* enzymes (New England Biolabs) and the purified digested PCR products and linearized vector were ligated with Quick Ligation™ Kit (New England Biolabs) to obtain pAM_*slo* plasmid DNA. The mix of 10 µg of pAM_*slo* plasmid DNA and 100 µL of electrocompetent 3348Δ*slo* cells was incubated on ice for 30 min, then transferred into an ice-cold 0.1 cm cuvette, electroporated at 1.8 kV with 400 Ω at 25 µF with a MicroPulser Electroporation Apparatus (BioRad), and incubated on ice for 5 min. Bacteria were then grown with 0.9 mL of THYE with 250 mmol/L of sucrose for 2 h at 37°C with 5% CO_2, centrifuged for 10 min at RT at 900 x *g* and grown on TSA plates with 5% sheep blood and 10 µg/mL chloramphenicol overnight at 37°C with 5% CO_2. Clones were screened by PCR. All *S. pyogenes* isolates were grown at 37°C with 5% CO_2 in Todd–Hewitt medium supplemented with 0.5% yeast extract (THY, Difco), or on Tryptic-soy agar (TSA) plates with 5% sheep blood, except for 3348Δ*slo*(pAM_*slo*) as stated above.

Histomorphological analysis and immunofluorescence on FFPE sections. Immunohistochemical staining (IHC) was performed in either Discovery XT or Discovery Ultra automated immunostainer (Ventana/Roche) on FFPE tonsil sections. For each immunostaining, immunoglobulins of the corresponding isotype were used as a negative control. For neutrophil detection, 4-µm sections were deparaffinized, then underwent heat-mediated antigen retrieval using TRIS-based buffer (Ultra CC1, Roche), and were then incubated for16 min at 37°C with ready-to-use Confirm anti-CD15 mouse MAb (Roche); detection was performed with anti-mouse alkaline phosphatase-conjugated secondary antibody (Ultra Map, Roche) for 20 min at 37°C followed by ChromoRed Map (Roche) detection system. Counterstain was performed with hematoxylin. For *S. pyogenes* detection, 4-µm deparaffinized sections underwent antigen retrieval as above. For IHC, sections were then incubated overnight at room temperature (rt) with a 1:200 dilution of home-made rabbit polyclonal anti-*S. pyogenes* whole cell antibody. Detection and counterstain were performed as above. For immunofluorescence, sections were saturated with 3% BSA in Phosphate Buffered Saline (PBS) and then were incubated with rabbit anti-*S. pyogenes* for 2h at RT; detection was performed with Alexa Fluor® 488 F(ab`)2 fragment of goat anti-rabbit IgG (H+L) for 1h at rt.

IHC images were acquired either by Mirax Scan 150 (Zeiss) slide scanner equipped with 40 x lens, or by Leica DM5500B microscope equipped with differential interference contrast (DIC) optics. Fluorescence and DIC images were acquired with Zeiss Axio Imager Z2 microscope, equipped with epifluorescence and DIC optics, and merged using Axio Vision software. Germinal centre (GC) grade (haematoxylin-eosin, HE) and neutrophil infiltrate level (HE and anti-CD15 IHC) were determined in sections of 34 FFPE palatine tonsils.

Immunofluorescence on frozen sections. 5 µm-thick tonsil slices were fixed with 2% formaldehyde, 60 mmol/L dibasic Na Phosphate pH 9.15, and 14 mmol/L monobasic Na Phosphate pH 7.4 in ultrapure water for 15 min, at rt; sections were washed with wash solution (0.5% BSA, 1% Saponin in PBS pH 7.4), and saturated with 10% horse serum in wash solution for 15 min, rt; after one wash, mouse monoclonal anti-TLR4 antibody with rabbit polyclonal anti-SLO or mouse polyclonal anti-*S. pyogenes* with rabbit polyclonal anti-SLO or mouse polyclonal anti-*S. pyogenes* alone were added to the specimens for 45 min at rt; after three washes, sections were saturated as above, then washed once and detected respectively with goat anti-rabbit Alexa Fluor® 568 plus goat anti-mouse Alexa Fluor® 488, or with goat anti-rabbit Alexa Fluor® 488 plus goat anti-mouse Alexa Fluor® 568, or with goat anti-mouse Alexa Fluor® 488 (Life Technologies) for 30 min at rt in the dark; samples were then washed twice in wash solution, once in PBS, and mounted with ProLong® Gold Antifade Reagent with DAPI (Life Technologies). Negative control slides without primary antibodies were prepared for each staining. Slides were observed on LSM 700 confocal laser scanning microscope (Zeiss).

Western blot assay. We performed western blot with mouse polyclonal anti-SLO on cell-free *S. pyogenes* supernatants harvested at A_{600} = 0.2, 0.4, and 0.8 from all *S. pyogenes* strains isolated in OSAS tonsils to verify SLO *in vitro* production; the supernatants were concentrated by trichloroacetic acid (TCA) protein precipitation [5], and the samples obtained were run as previously described [6].

Purification of human peripheral blood mononuclear cells (PBMCs). PBMCs from healthy volunteers from the San Giuseppe General Hospital (Empoli, Italy) were purified as described with minor adaptations [7]. Briefly, heparinised blood was mixed with a double volume of PBS and separated by Ficoll-paque, to obtain mononuclear cells which were washed in HBSS and incubated on ice for 2 min with 1X red blood cell (RBC)

lysis buffer. Cells were washed, filtered with 70 µm-cell strainers and counted. The cysteinyl leukotriene production assay was performed on PBMCs as described above.

Supplementary References S4

1. Bensi G*, et al.* Multi high-throughput approach for highly selective identification of vaccine candidates: the Group A Streptococcus case. *Molecular & cellular proteomics : MCP* **11**, M111 015693 (2012).

2. Murray PR, Masur H. Current approaches to the diagnosis of bacterial and fungal bloodstream infections in the intensive care unit. *Critical care medicine* **40**, 3277-3282 (2012).

3. Buccato S*, et al.* Use of Lactococcus lactis expressing pili from group B Streptococcus as a broad-coverage vaccine against streptococcal disease. *The Journal of infectious diseases* **194**, 331-340 (2006).

4. Chiarot E*, et al.* Targeted amino acid substitutions impair streptolysin O toxicity and group A Streptococcus virulence. *mBio* **4**, e00387-00312 (2013).

5. Rosch JW, Vega LA, Beyer JM, Lin A, Caparon MG. The signal recognition particle pathway is required for virulence in Streptococcus pyogenes. *Infection and immunity* **76**, 2612-2619 (2008).

6. Becherelli M*, et al.* Protective activity of the CnaBE3 domain conserved among Staphylococcus aureus Sdr proteins. *PloS one* **8**, e74718 (2013).

7. Pynaert G, Grooten J, van Deventer SJ, Peppelenbosch MP. Cysteinyl leukotrienes mediate histamine hypersensitivity ex vivo by increasing histamine receptor numbers. *Mol Med* **5**, 685-692 (1999).
